# Male Infertility and Oxidative Stress: A Focus on the Underlying Mechanisms

**DOI:** 10.3390/antiox11020306

**Published:** 2022-02-02

**Authors:** Robert John Aitken, Joël R. Drevet, Aron Moazamian, Parviz Gharagozloo

**Affiliations:** 1 Priority Research Centre for Reproductive Science, Discipline of Biological Sciences, School of Environmental and Life Sciences, College of Engineering Science and Environment, University of Newcastle, Callaghan, NSW 2308, Australia; 2 Hunter Medical Research Institute, New Lambton Heights, NSW 2305, Australia; 3 GReD Institute, INSERM U1103-CNRS UMR6293—Université Clermont Auvergne, Faculty of Medicine, CRBC Building, 28 Place Henri Dunant, 63001 Clermont-Ferrand, France; joel.drevet@uca.fr (J.R.D.); aron.moazamian@celloxess.com (A.M.); 4 CellOxess LLC, Ewing, NJ 08628, USA; parviz.gharagozloo@celloxess.com

**Keywords:** spermatozoa, oxidative stress, reactive oxygen species, sperm mitochondria, NADPH oxidase, amino acid oxidase, antioxidants

## Abstract

Reactive oxygen species (ROS) play a critical role in defining the functional competence of human spermatozoa. When generated in moderate amounts, ROS promote sperm capacitation by facilitating cholesterol efflux from the plasma membrane, enhancing cAMP generation, inducing cytoplasmic alkalinization, increasing intracellular calcium levels, and stimulating the protein phosphorylation events that drive the attainment of a capacitated state. However, when ROS generation is excessive and/or the antioxidant defences of the reproductive system are compromised, a state of oxidative stress may be induced that disrupts the fertilizing capacity of the spermatozoa and the structural integrity of their DNA. This article focusses on the sources of ROS within this system and examines the circumstances under which the adequacy of antioxidant protection might become a limiting factor. Seminal leukocyte contamination can contribute to oxidative stress in the ejaculate while, in the germ line, the dysregulation of electron transport in the sperm mitochondria, elevated NADPH oxidase activity, or the excessive stimulation of amino acid oxidase action are all potential contributors to oxidative stress. A knowledge of the mechanisms responsible for creating such stress within the human ejaculate is essential in order to develop better antioxidant strategies that avoid the unintentional creation of its reductive counterpart.

## 1. Introduction

The notion that oxidative stress is a major factor in the aetiology of male infertility has a long history stretching back to studies performed in the 1920s, when the impact of vitamin E deficiency on normal testicular function became apparent [1]. Pioneering studies conducted by Thaddeus Mann and colleagues at the University of Cambridge extended this concept when they elegantly demonstrated the damaging impact of lipid peroxidation on normal sperm function [2,3]. They were the first to point out the vulnerability of mammalian spermatozoa to peroxidative damage because of their high cellular content of polyunsaturated fatty acids, and the first to identify the particular vulnerability of plasmalogen to this process. They also highlighted the ability of fatty acid peroxides to rapidly, and permanently, arrest the motility of human spermatozoa [4], thereby opening the door to a pathophysiological mechanism that is now recognized as a major cause of defective sperm function in all species examined, from sea urchins to man [5].

The relevance of this phenomenon to human infertility was emphasized by two independent publications appearing in 1987. In the first of these articles, human spermatozoa were shown to be capable of generating reactive oxygen species (ROS) in a manner that could be stimulated by calcium and exhibited a profound negative correlation with the fertilizing ability of these cells in a sperm–oocyte fusion assay [6]. In the second, human spermatozoa were shown to undergo spontaneous lipid peroxidation when incubated in vitro, due to the production of both a superoxide anion (O_2_^•−^) and hydrogen peroxide (H_2_O_2_). The latter was held to largely be the result of O_2_^•−^ dismutation in response to varying levels of superoxide dismutase within individual sperm populations [7]. Subsequent studies established that it is the H_2_O_2_ generated by these cells that has the greatest impact on sperm function [8], although the rapid interconversion of ROS should be acknowledged in this context. In addition to H_2_O_2_ and O_2_^•−^, spermatozoa also generate another free radical, nitric oxide (NO), as well as other powerful oxidants such as peroxynitrite (ONOO-) or hypochlorous acid (HOCl), as well as peroxyl radicals (ROO^•^), alkoxyl radicals (RO^•^), and organic hydroperoxides (ROOH), any, and all of which, can be damaging to spermatozoa when generated in excess.

Further analysis of the impact of ROS on sperm function also revealed that these reactive oxygen metabolites were not just harbingers of cellular dysfunction and death. They are also essential for normal sperm function, stimulating elements of both sperm capacitation and fertilization in a wide variety of animal and plant species [9,10], suggesting a prolonged evolutionary conservation of the underlying mechanisms. ROS are thus a two-edged sword as far as spermatozoa are concerned. They are needed in small quantities to drive various elements of sperm function, but when generated in excess, they rapidly overwhelm the limited antioxidant defences offered by these cells and induce a state of oxidative stress [11,12]. Oxidative stress in the male germ line has become an extensive field of investigation covering the promotion and suppression of sperm production and function, as well as the genetic and epigenetic changes in the germ line that impact the health and wellbeing of the offspring [11,13]. This review is not intended to cover all these aspects, but rather constitutes an in-depth assessment of the causes of oxidative stress in the male germ line and potential strategies for addressing this pathology in a therapeutic context.

## 2. Sources of ROS in the Human Ejaculate

### 2.1. Leukocytes

Every single human semen sample is contaminated with leukocytes, comprised largely of peroxidase-positive eosinophils and neutrophils, macrophages, and a lesser number of B- and T- lymphocytes [14]. The actual number of leukocytes, and the precise subsets represented in the human ejaculate, vary considerably between individuals. Using monoclonal antibodies, the average number of leukocytes in unselected semen samples was found to be around (mean ± standard error) 39.2 ± 9.0 × 10^4^ per mL, comprised mostly of granulocytes (39.7 ± 7.6 × 10^4^ per mL), with a small number of macrophages/monocytes (3.8 ± 2.0 × 10^4^ per mL), B-cells (3.8 ± 0.9 × 10^4^ per mL), and T-cells (6.1 ± 1.6 × 10^4^ per mL) [15]. Pathological levels of leukocytic infiltration, leading to a state of leukocytospermia, is said to occur when the number of leukocytes becomes significantly elevated to a value above 1 × 10^6^ per mL. There is very little substance to this threshold; it is just a convenient contrivance for the classification of patients. Nevertheless, it has served its purpose in highlighting cases of infertility where the presence of leukocytes seems to be a key feature of the pathological landscape, even though the clinical significance of leukocytospermia has been mired in controversy for a variety of reasons.

For one thing, there is very little agreement as to how leukocytes can be identified and quantified. It is clear that peroxidase, esterase, and elastase assays do not provide consistent data [14], while the optimal method, using antibodies against the common leukocyte antigen CD45, has only been employed in a minority of studies. The measurement of pro-inflammatory cytokines may be a convenient solution to this problem, but again, there is very little agreement on this issue. Interleukin-8 (IL-8) has been found to correlate with sperm DNA damage and sperm vitality in certain studies, but no other cytokine [16]. Alternatively, other studies found significantly increased levels of IL-2, IL-4, IL-6, IFN-γ, and, particularly, TNF-α in human semen in association with leukocytospermia [17,18,19,20]. These, and other seminal cytokine-like molecules such as resistin [21], are clearly indicative of a proinflammatory state and are intimately associated with the induction of leucocyte infiltration; however, there is little evidence to suggest that cytokines are directly responsible for any changes in sperm functionality [22]. The possible exception is TNF-α, which, in vitro, has been shown to suppress sperm motility, collapse mitochondrial membrane potential, and increase levels of DNA fragmentation in concert with the induction of sperm apoptosis [23,24]. The clinical relevance of these in vitro studies is questionable, however, because there seems to be no correlation between seminal TNF-α concentrations and sperm quality [22], although weak negative correlations with sperm motility have been observed in certain studies [25,26]. The one consistent factor linking leukocytospermia and defective sperm function is oxidative stress. The infiltration of leukocytes into the male tract has been associated with increases in biomarkers of peroxidative damage such as malondialdehyde [16] and the augmented generation of seminal ROS [27], as well as indicators of oxidative stress in spermatozoa including DNA fragmentation [16], 8OHdG formation in the sperm nucleus [28], and reduced sperm motility [29].

Whether or not leukocytospermia is associated with defective sperm function will depend on the numbers of leukocytes involved and the leukocyte subsets that are present. Assuming that phagocytic granulocytes and macrophages are largely responsible for the observed increase in ROS generation [27], then much will also depend on how the leukocytes were activated, when they were activated, and, most important of all, where they were activated. The ‘how’ question is a particular mystery. It is known that neither bacterial nor viral infection is consistently correlated with leukocytospermia [30,31,32]. So why does leukocytic infiltration occur? Presumably, a variety of pro-inflammatory states generated by such conditions as varicocele [33], spinal cord injury [34], cigarette smoking [35], obesity [36], and infection, including COVID-19 [37], promote the local expression of cytokines such as IL-8 that, in turn, stimulate an influx of leucocytes into the male reproductive tract [36]. If, as is generally assumed, these cells enter the ejaculate via the secondary sexual glands, then the first time that leucocytes will come into contact with the spermatozoa will be at the moment of ejaculation, when the spermatozoa will be protected by a pool of powerful and diverse antioxidants in the seminal plasma [2,5,11]. Alternatively, if the leukocytic infiltration is secondary to orchitis or epididymitis, then the implications for sperm function are significantly greater since the leukocytes will have had more time to overwhelm the local antioxidant protection offered by the male reproductive tract and damage the spermatozoa. The fact that vasectomized samples contain few leukocytes [38] has been taken as evidence for the importance of the epididymis as a point of entry for infiltrating leukocytes. However, without a knowledge of the pre-vasectomy levels of leukocytospermia, it is hard to be confident as to how, where, and why this condition arises, and, thus, its clinical significance remains uncertain.

The one element of this equation that does appear to be consistent is that the oxidative stress associated with leukocytospermia is involved in the suppression of sperm movement [39,40,41] as well as the induction of DNA damage [16]. Furthermore, the beneficial effects of antioxidants on sperm movement, including hyperactivation, suggests that such associations may be causative in nature, not merely correlative [42]. The means by which leukocytospermia reduces sperm counts and morphology, as observed in one study [40], is more puzzling. It is possible that the epididymis is effective in removing morphologically abnormal cells from the tract as a quality control measure [43]. Therefore, mysteries persist. Up to a point, the antioxidants secreted by the male tract are capable of protecting the spermatozoa from serious damage [15], but, eventually, even these guardians of the germ line become overwhelmed and the functionality of the spermatozoa is compromised [44,45]. In vitro, the situation changes dramatically because seminal plasma antioxidants are no longer available to protect the spermatozoa when these cells are physically isolated and suspended in culture medium. As a result, even low levels of leukocyte contamination in washed sperm suspensions are able to suppress the fertilizing potential of these cells in vitro [46], even when ICSI is used as the mode of insemination [47].

### 2.2. Spermatozoa

#### 2.2.1. Sperm Mitochondria

The second source of ROS in the ejaculate is the spermatozoa themselves. It has been known since 1946 that spermatozoa are capable of generating ROS [48], although the underlying mechanisms still have to be resolved in detail. In this context, clear contributors to the redox status of the male germ line are the sperm mitochondria [49,50]. In certain species, such as the horse, the production of ATP by their mitochondria is so central to their metabolism that ROS generation by these organelles is positively correlated with the sperm motility and the ability of these cells to establish a pregnancy in vivo [49]. Human spermatozoa are not so inclined. In our own species, the spermatozoa rely heavily on glycolysis to meet their energy needs, and ROS generation by the mitochondria is a pathological change associated with a loss of sperm motility [50] and the induction of sperm apoptosis [51]. In this situation, the impaired control of electron transport within the inner mitochondrial membrane leads to the leakage of these negatively charged subatomic particles that are swept up by the universal electron acceptor, oxygen, to generate O_2_^•−^. The latter then rapidly dismutates, under the influence of superoxide dismutase, to generate the powerful oxidant, H_2_O_2_ [7,52]. H_2_O_2_ is both a blessing and a curse as far as the functional competence of human spermatozoa is concerned. When generated in small amounts, H_2_O_2_ has been shown to have a positive impact on sperm capacitation and sperm–oocyte fusion via a variety of mechanisms, including the suppression of tyrosine phosphatase activity, the stimulation of cAMP generation, and the induction of cholesterol efflux from the plasma membrane [11,53,54,55,56]. When generated in excess, the antioxidant defences of this vulnerable cell type are rapidly overwhelmed and the spermatozoa suffer a catastrophic loss of functionality together with a significant increase in levels of sperm DNA damage [8,54,57]. Electron leakage seems to occur at complexes I and III, with the most damaging being the release of ROS on the matrix side of the inner mitochondrial membrane at complex I [50]. Exactly what disturbs the orderly flow of electrons along the mitochondrial electron transport chain to cytochrome oxidase is not known with certainty; however, some leads are emerging as discussed below.

**Electrophilic aldehydes.** Certainly, one element of this process is the adduction of proteins involved in mitochondrial electron transport by lipid aldehydes, such as 4-hydroxynonenal (4-HNE) or acrolein, generated during the lipid peroxidation process [58]. Viewed in this light, the generation of ROS by the sperm mitochondria plays an important role in amplifying the amount of cellular damage instigated via the induction of oxidative stress. Once the lipid peroxidation process has been initiated, the aldehydes generated as a result of this process will form adducts with proteins, including several that are involved in the orchestration of mitochondrial electron transport. This leads to electron leakage, O_2_^•−^ generation, and the precipitation of yet more lipoperoxidative damage in a self-perpetuating spiral. Spermatozoa are particularly vulnerable to this process because they are so enriched in the polyunsaturated fatty acids that drive the lipid peroxidation process [2,59]. The presence of multiple, unsaturated bonds in these fatty acids renders the bis allylic carbons vulnerable to hydrogen abstraction and the creation of a carbon-centred radical. The latter then binds with oxygen to generate peroxyl (ROO^•^) and alkoxyl (RO^•^) radicals that, to stabilize, abstract hydrogen atoms from adjacent lipids in a propagative cycle. These chemical reactions culminate in the generation of small molecular mass electrophilic lipid aldehydes such as 4-HNE, acrolein, and malondialdehyde, which are, to varying degrees and via various mechanisms, damaging to spermatozoa [58,59,60,61], with the mitochondria being a major target. It has been estimated that around 30% of 4-HNE adducted proteins are located in the mitochondria, and proteomic analysis has revealed that many of these proteins are constituents of the electron transport chain, including the ATP synthase subunit β (ATP5B), succinate dehydrogenase [ubiquinone] flavoprotein subunit (SDHA), and NADH dehydrogenase [ubiquinone] iron–sulfur protein 2 (NDUFS2) [62].

The ability of 4-HNE to adduct these proteins and stimulate mitochondrial ROS generation is also reinforced by the tendency of this aldehyde to form adducts with aldehyde dehydrogenase (ALDH), a key enzyme in the protection of spermatozoa against oxidative stress. Using stallion spermatozoa as a model, strong correlations have been detected between ALDH expression and various aspects of sperm motility, including the percentage of progressive and rapidly motile spermatozoa. Prolonged incubation of these cells in vitro has also revealed highly significant correlations between progressive motility loss, 4-HNE accumulation, and ALDH expression. Moreover, the pharmacological inhibition of ALDH resulted in a spontaneous increase in 4-HNE levels in viable cells and a corresponding decrease in total and progressive motility over 24 h [63]. This inhibition appears to result from the formation of a 4-HNE–cysteine adduct at the ALDH2 active site [64], and is suggested to be an important component of the way in which lipid peroxidation cascades impact sperm function. Interestingly, no studies have been conducted to date on the role of ALDH in the aetiology of human male infertility; however, given its importance in other cellular systems as well as the spermatozoa of other species, it is likely to be an important factor in the cascade of changes that mediate the impact of oxidative stress on human sperm function.

In addition to the lipid aldehydes generated as a result of lipid peroxidation, a variety of other electrophiles are known to disturb the orderly flow of electrons along the mitochondrial electron transport chain and generate ROS. For example, the anti-fertility agent gossypol [65], the vitamin K mimetic menadione [66], and the homocysteine cyclic congener homocysteine thiolactone [67] are all examples of powerful electrophiles that modify mitochondrial proteins and trigger ROS generation through the initiation of a lipoperoxidative cascade.

**Figure 1:**
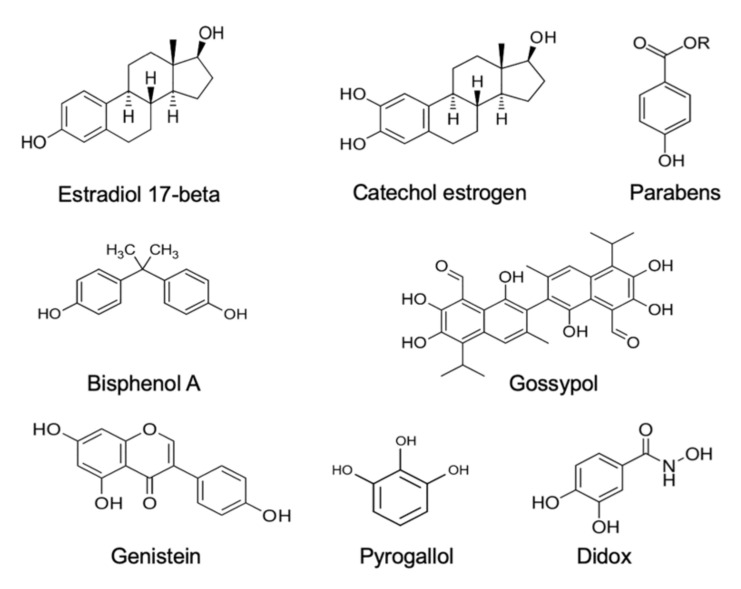
Examples of some of the estrogenic compounds that have been found to induce mitochondrial ROS generation in purified populations of human spermatozoa. ROS generation generally occurs via the redox cycling on these compounds within the mitochondria.

**Estrogens.** Xenobiotics that are classified as environmental estrogens also trigger mitochondrial ROS generation in human spermatozoa. Included in this group of compounds are parabens, a widespread preservative used on the cosmetics industry [68], the above-mentioned gossypol, a potential male contraceptive agent [65], and a variety of phenolic compounds that are generally held to possess antioxidant properties, such as pyrogallol and didox [65], as well as powerful synthetic estrogens such as diethylstilbestrol [65,69]. In addition, bisphenol A, a chemical produced in large quantities for use in the production of polycarbonate plastics and epoxy resins [70], has been found to stimulate mitochondrial ROS generation, but only when high doses (>300 µM) and prolonged exposure periods (4 h) were used. Similarly, the parent estrogen (estradiol-17β) has been found to induce mitochondrial ROS generation in high doses [71], possibly after being converted to catechol estrogens that are much more redox active than estradiol-17β and constitute powerful inducers of mitochondrial ROS generation by human spermatozoa, as well as other cell types [66,69,72]. All of these oestrogen-like compounds that are capable of eliciting mitochondrial ROS generation by human spermatozoa share a key structural feature, which is a hydroxylated aromatic ring (Figure 1).

Whether ROS generation from the mitochondria in the presence of such compounds is associated with oxidative stress and a loss of sperm function depends on where in the electron transport chain the induction of ROS generation occurs, as well as the level of redox activity. Many of the antioxidant polyphenols that are actually known to be beneficial for sperm function induce ROS generation at complex III, from whence the toxic oxygen metabolites are released into the intramembranous space and, ultimately, the cytoplasm, with minimal induction of cellular damage [50,65]. Damage occurs when ROS are released into the mitochondrial matrix, whereupon they induce lipid peroxidation and the total disruption of sperm function due to the formation of toxic electrophiles, such as 4-HNE, that form adducts with both DNA and a range of functionally important proteins. In this context, catechol estrogens are thought to elicit ROS generation on the matrix side of the inner mitochondria membrane and to have a particularly devastating impact on sperm function as a consequence [66,69]. The speed with which catechol estrogens stimulate mitochondrial ROS in spermatozoa suggests a redox-cycling mechanism [73]. For catechol estrogens to redox cycle, they have to be metabolised to the corresponding semiquinone via oxidoreductases, including P450 isoforms that are located on the inner mitochondrial membranes with their active sites facing the mitochondrial matrix [74]. The fact that not all phenolic compounds are capable of eliciting ROS formation [65,69] suggests that the redox cycling oxidoreductases in the sperm mitochondrial matrix exhibit some substrate specificity. The much weaker effect of estradiol-17β may also reflect the ability of this compound to increase the activity of complex IV of the electron transport chain, improving mitochondrial respiration and ATP production [75]. In doing so, this natural hormone may increase mitochondrial redox activity sufficiently to create spontaneous electron leakage and mitochondrial ROS formation. The available data therefore suggests that estrogenic compounds in the water we drink, the food we eat, and the environment we inhabit could all be contributing to the oxidative stress experienced by the male germ line in modern industrialized societies, leading to a loss of sperm function and an increase in oxidative DNA damage.

Interestingly, data is gradually emerging suggesting a relationship between the global decline in sperm counts and a secular decline in circulating testosterone levels. Thus, in Nordic countries [76], the USA [77], and Israel [78], evidence has been generated indicating that testosterone levels have been declining since the 1970s, in concert with the decline in sperm counts. The decline in testosterone levels might, in turn, be driven by a rise in oestrogen exposures from multiple sources [79]. Since environmental estrogens such as bisphenol A [80] and parabens [81], as well as estradiol itself [82], are known to not only suppress testosterone levels, but also induce testicular oxidative stress, it is possible that the pollution of our environment with estrogenic compounds is compromising male reproductive function via a variety of mechanisms. The ability of antioxidants to reverse the reproductive pathology associated with the administration of such exogenous estrogens [80,81] emphasises just how important oxidative stress is in this overall situation.

**Polyunsaturated fatty acids.** In light of the above, it is possible that chronic exposure to oestrogen-like compounds encountered in the environment, or as a consequence of lifestyle and/or occupation, can enhance mitochondrial ROS generation by human sperm mitochondria. Another group of compounds that are involved in the stimulation of mitochondrial ROS generation by human spermatozoa are polyunsaturated fatty acids (PUFA). Exposure of these cells to free, unesterified, unsaturated fatty acids elicits a powerful mitochondrial ROS response in human spermatozoa; the more unsaturated the fatty acid, the more reactive oxygen metabolites are generated [83]. Thus, the major PUFAs in human spermatozoa, arachidonic and docosahexaenoic acids, are capable of inducing significant mitochondrial ROS formation in these cells, promoting peroxidative damage and generating both a loss of sperm motility and an increase in oxidative DNA damage [83,84,85]. Saturated fatty acids, unsaturated fatty acid esters, or a variety of other amphiphiles were all ineffective in this regard [83]. As such, while the amphipathic properties of the PUFA are clearly important, there are other structural constraints defining the ability of these molecules to trigger sperm mitochondrial ROS generation. Importantly, an analysis of the PUFA content of untreated human spermatozoa revealed a positive correlation with spontaneous mitochondrial ROS generation [84]. Thus, defective human spermatozoa are characterized by an abnormally high content of fatty acids that, in their unesterified, unsaturated form, promote ROS generation by sperm mitochondria, creating a state of oxidative stress and a concomitant loss of functional competence. There are important implications in these studies for the impact of fat-rich, Westernized diets on the biochemical composition and function of human spermatozoa through the capacity of unesterified PUFA to promote oxidative damage in the male germ line [86].

**Electromagnetic radiation.** Various types of electromagnetic energy, including heat, visible light, and radiofrequency radiation, are known to trigger the generation of mitochondrial ROS in populations of mammalian spermatozoa. The impact of radiofrequency electromagnetic radiation (RF-EMR) on human sperm function was first suggested in the context of mobile phone usage, which was found to have a damaging impact on both sperm motility and DNA integrity [87,88,89]. De Iuliis et al. [90] subsequently demonstrated that the loss of sperm function observed following RF-EMF exposure was related to the induction of mitochondrial ROS generation in human spermatozoa. These observations have been confirmed in animal models [91,92,93] and, for the first time, suggest a mechanism by which such low frequency radiation might exert a biological effect. Subsequently, a plethora of studies have demonstrated the ability of RF-EMF to trigger mitochondrial ROS generation in male germ cells and simultaneously inhibit the cells’ antioxidant defences [94]. The exact mechanism by which such low frequency radiation stimulates mitochondrial ROS generation in spermatozoa is currently unknown, but may, as suggested above, involve an increase in electron flux along the mitochondrial electron transport chain followed by electron leakage and the reduction of ground state oxygen to O_2_^•−^.

Heat is another form of electromagnetic energy that can have adverse impacts on sperm function through the stimulation of mitochondrial ROS generation both in vitro and, in the case of varicocele, in vivo [90]. Furthermore, exposure of whole animals to elevated ambient temperatures can also stimulate mitochondrial ROS generation, which secondarily enhances the levels of oxidative DNA damage and fragmentation in the germ line [95,96]. Again, the mechanism underpinning the impact of heat is not known, but an initial acceleration of electron flux through the mitochondrial electron transport chain, accompanied by electron leakage and ROS generation, seems like a reasonable hypothesis.

Interestingly, visible light is also thought to increase mitochondrial ROS generation in spermatozoa via the stimulation of photosensitive pigments (probably flavins) in these cells [97]. The wavelength of the light seems be an important element in the cascade of changes that lead to mitochondrial ROS generation. Thus, a brief (<5 min) exposure to red light (620–630 nm) was found to stimulate sperm motility and ROS generation by equine and, to a much less extent, ram spermatozoa [98]. Similarly, red light (maximum energy at 600 nm) has been found to stimulate ROS generation in populations of human spermatozoa, which responded by exhibiting a burst of hyperactivated motility and associated shifts in intracellular calcium and cAMP [99]. Other studies have found that blue light in the 400–500 nm range is capable of activating ROS generation in sperm suspensions [100]. Blue light has also been found to induce NO formation in spermatozoa, although, as in most such studies, the sub-cellular source of ROS/NO was not identified [101]. Visible light is known to induce mitochondrial ROS in other cell types via mechanisms that involve an increase in intramitochondrial calcium levels [102]. Indeed, in the oligodendrocyte cell line MO3.13, this relationship between intramitochondrial calcium levels and mitochondrial ROS appears to be causative [103], while in hepatocytes, calcium-dependent mitochondrial ROS generation is thought to be instrumental in eliciting the opening of the mitochondrial permeability transition pore that precedes cell death [104]. As such, in human spermatozoa, there may well be a complex interplay between the exposure to visible light, elevated intracellular calcium, mitochondrial permeability transition pore opening, and ROS generation [105]. The major take-home message from these studies is that carefully controlling the exposure of mammalian spermatozoa to visible light is important in maintaining the functional integrity of these cells during assisted conception procedures [105].

If the intensity of the radiation is increased to include UVC irradiation (200–280 nm), then the impact on sperm function is entirely negative, generating a diminution of sperm motility, viability, and, concomitantly, an increase of the level of lipid peroxidation. The observed effects of the UVC irradiation were prevented by the addition of the antioxidant butylated hydroxy toluene, indicating that the effects are mediated via the generation of ROS and oxidative stress [106]; however, in this instance, the mitochondria are not known to be involved.

**Apoptosis.** Mitochondrial ROS generation is also a distinctive feature of spermatozoa as they enter the intrinsic apoptotic pathway. Spermatozoa are capable of undergoing a truncated form of apoptosis characterized by rapid motility loss, mitochondrial ROS generation, caspase activation in the cytosol, cytoplasmic vacuolization, and oxidative DNA damage [107]. In many ways, death via the intrinsic apoptotic cascade is the default destiny for this cell type as all ejaculated spermatozoa are preordained to become senescent and die except, of course, the cell that successfully achieves fertilization, which are rewarded with a certain measure of immortality.

The signal transduction pathway that prevents spermatozoa from entering the intrinsic apoptotic cascade is phosphoinositide-3 kinase (PI3 kinase) and AKT1 (serine/threonine kinase 1). As long as these enzymes are phosphorylated and active, the spermatozoa are prevented from undergoing apoptosis and will not become senescent. However, if PI3 kinase activity is suppressed with compounds such as wortmannin, then the spermatozoa rapidly enter the intrinsic apoptotic cascade [107]. There are a variety of prosurvival factors present in the male and female reproductive tracts that are designed to keep spermatozoa in a viable, functional state by maintaining PI3 kinase activity in vivo, including insulin, prolactin, or angiotensin 1–7 [108,109,110]. PI3 kinase phosphorylates AKT and, as long as the latter is phosphorylated, downstream targets of this kinase, such as the apoptosis regulator BCL2-associated-agonist-of-cell-death (BAD), are also phosphorylated. Phospho-BAD forms a heterodimer with its 14-3-3 keeper protein, leaving Bcl-2 free to inhibit Bax-triggered apoptosis, thereby maintaining spermatozoa in a viable motile state free from ROS generation. However, if PI3K activity is disrupted or lost, then AKT and its downstream target, BAD, become dephosphorylated, allowing the latter to escape from 14-3-3 to form a heterodimer with Bcl-2 and Bcl-xL, thereby inactivating these regulators and, consequently, allowing Bax/Bak-triggered apoptosis. As soon as the latter proteins permeabilize the outer mitochondrial membrane, ROS are generated that advance the apoptotic process and accelerate the functional demise of the spermatozoa.

#### 2.2.2. NADPH Oxidases

Another class of enzymes thought to be involved in the production of ROS by human spermatozoa are the NADPH oxidases (NOX). This has been a hotly contested area that was finally laid to rest when Banfi and colleagues discovered that these cells possess an unusual NOX, named NOX5 [111]. The clinical significance of NOX5 is indicated by its high level of expression in the spermatozoa of asthenozoospermic males, in association with the enhanced generation of both O_2_^•−^ and H_2_O_2_ and DNA damage [112]. NOX5 expression has also been shown to be elevated in cases of teratozoospermia [113].

NOX5 is unusual in that it possesses EF-hand motifs that render the enzyme responsive to calcium. This feature explains why reagents that elevate intracellular calcium levels, such as divalent cation ionophores (A23187 and ionomycin) and progesterone, have been found to be capable of triggering ROS generation by human spermatozoa via mechanisms that can be blocked by DPI (diphenylene iodonium), SOD (superoxide dismutase), zinc, and the calcium chelator BAPTA (1,2-bis(o-aminophenoxy)ethane-N,N,N′,N′-tetraacetic acid) [114,115,116,117]. Furthermore, ROS production can be activated by phorbol esters that activate protein kinase C, a known regulator of NOX activity [118].

A key feature of NOX5 is its dependence on the HvI voltage-regulated proton channel and the tyrosine kinase c-Abl [116]. The HvI proton channel, is required for optimal O_2_^•−^ production because it prevents the cytoplasmic acidification (proton generation) that inevitably follows NADPH oxidation:

NADPH + 2O_2_ = NADP+ + 2O_2_^•−^ + H+

     NADPH oxidase superoxide proton

**Figure 2:**
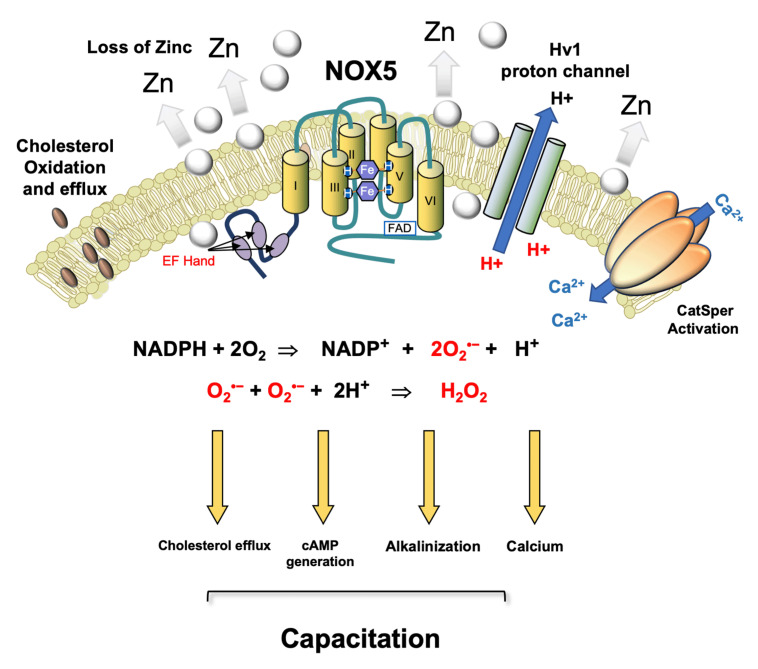
Postulated role for NOX 5 in driving sperm capacitation in human spermatozoa. This is a calcium sensitive NADPH oxidase that generates both the superoxide anion and protons. A possible pathway for human sperm capacitation begins with a reduction in ambient zinc concentrations as they move from the high zinc environment provided by seminal plasma to the relatively low zinc environment offered by the female reproductive tract. Since zinc inhibits the Hv1 proton channel, this change favours cytoplasmic alkalinization. As the spermatozoa ascend the female tract and prepare for fertilization, they are stimulated by progesterone which, in combination with the independent increase in cytoplasmic pH induced by Hv1 activation, triggers an influx of calcium via the low-voltage-dependent calcium channel, CatSper. CatSper-dependent calcium influx activates NOX5, generating O_2_^•−^ which, along with bicarbonate and calcium, stimulates adenylyl cyclase and the production of cAMP. NOX5 also produces the protons required for optimal Hv1 activity. Furthermore, the intracellular conversion of O_2_^•−^ into H_2_O_2_ by superoxide dismutase consumes protons, thereby facilitating the alkalinization of the cells’ interior. The H_2_O_2_ generated by this dismutation reaction promotes cholesterol oxidation and efflux from the plasma membrane [56,120] and simultaneously suppresses tyrosine phosphatase activity, thereby facilitating the increase in tyrosine phosphorylation that accompanies capacitation. Thus, in a synergistic cooperation, Catsper, Hv1, and NOX5 control the primary drivers of capacitation including cholesterol efflux, cAMP generation, cytoplasmic alkalinization, and elevated intracellular free calcium. After Seredenina et al. [121].

The cytoplasmic alkalinization induced by the activation of this proton channel is thought to enhance calcium signalling in response to progesterone stimulation [117]. This may, in turn, reflect the fact that Catsper, the sperm specific calcium channel responsible for orchestrating the cellular influx of calcium following progesterone stimulation, is extremely pH dependent [119]. Thus, it is possible that NOX5, and the ROS generation it triggers, are key regulators of sperm function, shutting down tyrosine phosphatase activity, orchestrating cholesterol efflux from the plasma membrane, enhancing cAMP generation, and precipitating the cytoplasmic alkalinization needed to drive calcium influx via Catsper (Figure 2).

In cases where the spermatozoa are under oxidative stress because of excessive NOX activity, one of the causative factors may be the retention of large amounts of residual cytoplasm during spermiogenesis. The presence of such residual cytoplasm facilitates the generation of high levels of NADPH needed to fuel oxidase activity by virtue of the cytoplasmic enzyme glucose-6-phosphate dehydrogenase, which is a key regulator of the hexose monophosphate shunt responsible for regulating NADPH availability [122]. In addition, oxidative damage to the plasma membrane and/or redundant nuclear envelope in defective human spermatozoa may lead to the generation of high levels of intracellular calcium that would then further enhance NOX5 activity in a downward spiral, culminating in oxidative stress, apoptosis, and death.

NOX 5 is also known to be a key biochemical component of spermatozoa from other species. For example, evidence has been generated suggesting a key role for this enzyme in the redox regulation of sperm capacitation in the ram [123]. NOX5 has also been detected in canine and equine spermatozoa [124,125] and may well be involved in the redox regulation of sperm capacitation in these species [126,127]. The gene has also been detected in the genomes of an array of species from cattle to the gray mouse lemur and rhesus monkey, although a role in sperm capacitation has yet to be established for these species. Interestingly, there is strong evidence for capacitation being redox controlled in other species, such as the mouse [128], rat [129], and hamster [130], and yet none of these species possess the NOX5 gene in their genomes. In these species, other forms of NADPH oxidase (such as Duox 1 or 2 and Nox1, 3, or 4) may be redox active in the male germ line. Clearly, more work needs to be done to fully characterize the full array of NOX enzymes in the male germ line and their respective roles in the regulation of sperm function. Alternatively, these species may use an entirely different biochemical mechanism to generate the ROS that drive the capacitation process. In this context, there may also be an important role for oxidases that use aromatic amino acids, such as phenylalanine, tyrosine, and tryptophan, as their preferred substrate.

#### 2.2.3. Amino Acid Oxidases

The first enzyme that was ever shown to generate ROS in mammalian spermatozoa was an L-amino acid oxidase, which, more than 70 years ago, was recognised by Tosic and Walton [131] as the enzyme responsible for the depression of bovine sperm motility in the presence of egg yolk extenders. This enzyme generates significant quantities of H_2_O_2_ according to the equation:

L-α-amino acid + H_2_O + O_2_ = 2-oxocarboxylate + H_2_O_2_ + NH^4+^

Dead cells within the bovine ejaculate retain this enzyme [132] and respond to the aromatic amino acids in egg yolk by generating copious quantities of H_2_O_2_ because the disrupted plasma membrane allows this substrate access to the oxidase. As such, when bovine spermatozoa are exposed to high concentrations of aromatic amino acids, the dead cells generate ROS, which then impede the motility and fertilizing potential of live cells in their immediate vicinity. The same oxidase has been detected in equine [133], porcine [134], ovine [135], and human spermatozoa [136]. In the latter, oxidase activity is lost from non-viable cells, so the ability of dead cells to impact the viability of live cells with the same sperm suspension, as established for ungulate spermatozoa, does not apply in the human situation.

The physiological purpose of this oxidase appears to be related to the redox regulation of sperm capacitation. Stimulation of L-amino acid oxidase activity in human spermatozoa results in the induction of several hallmarks of capacitation, including tyrosine phosphorylation of the sperm flagellum and the concomitant activation of phospho-SRC expression. In addition, stimulation of this oxidase results in an increase in the levels of acrosomal exocytosis in both the presence and absence of progesterone stimulation via mechanisms that could be significantly reversed by the presence of catalase. Thus, it appears that this enzyme may be physiologically involved in stimulating the ROS production that, in turn, drives sperm capacitation [136]. This hypothesis has recently been born out in the mouse, where the genetic deletion of this L-amino acid oxidase gene has been shown to reduce male fertility in concert with a reduction in the amount of H_2_O_2_ generated by the spermatozoa and an impaired ability of the spermatozoa to acrosome react in response to the calcium signals generated by the ionophore, A23187 [137].

#### 2.2.4. Depletion of Antioxidants

In addition to the excessive generation of ROS, oxidative stress may also be driven by a deficiency in available antioxidant protection. While it is generally appreciated that defective semen quality is commonly associated with low levels of antioxidant protection in semen [12,138,139], it is always difficult to differentiate the cause and effect in such circumstances—did the lack of antioxidant protection induce a state of oxidative stress or did the excessive generation of ROS by spermatozoa, infiltrating leukocytes, or, in cases of obesity, adipocytes lead to the depletion of antioxidant equivalents from the semen?

In those circumstances, where the deletion of antioxidants is the primary cause of oxidative stress rather than a consequence, the antioxidant content of a patient’s diet is usually a key consideration [12,140]. For example, depletion of vitamin C from the diet leads to a dramatic increase in the levels of oxidative DNA damage in the spermatozoa, which can be significantly reduced by reintroducing vitamin C [141]. Zinc is another powerful antioxidant in the context of male reproduction and a deficiency of this element in the diet is also known to create oxidative stress in spermatozoa and impair male fertility [142,143]. Zinc concentrations in seminal plasma are significantly higher in normozoospermic donors than infertile patients and correlate positively with both sperm motility and count and negatively with malondiadelhyde, as a marker of oxidative stress [143]. In animal models, induced Zn deficiency has also been found to exacerbate diabetes-induced testicular oxidative damage and cell death [144]. From a clinical perspective, the administration of zinc to asthenozoospermic patients has been found to reduce levels of oxidative stress and improve DNA integrity in human spermatozoa [145].

Antioxidant depletion may also be observed when the metabolism of xenobiotics generates electrophilic derivatives that are subject to nucleophilic attack by compounds such as glutathione at rates that cannot be replenished. Codeine metabolism is an interesting case in point; the administration of this pharmaceutical agent leads to the formation of an electrophilic derivative, codeinone, that then leads to glutathione depletion and high levels of oxidative DNA damage in the spermatozoa [146,147]. Similarly, cytochrome P450 enzymes catalyse the metabolism of paracetamol to N-acetyl-p-benzoquinone imine, which then consumes glutathione in an attempt to neutralize this pernicious electrophile [148], causing oxidative damage to the testes and decreasing both sperm vitality and motility [149]. The oxidative stress associated with both active and passive smoking is also known to exhaust vitamins C and E levels in both blood and seminal plasma [150,151]. Methotrexate, a drug commonly used to treat inflammatory types of arthritis, is also known to induce toxicity via the depletion of glutathione [152] and is associated with testicular oxidative stress, the suppression of sperm motility, and the induction of sperm DNA damage [153,154]. In principle, any compound that becomes conjugated to glutathione by glutathione-S-transferase in the germ line has the potential to induce glutathione depletion and render these cells very vulnerable to oxidative stress, including the classical GSH-conjugating agents diethyl maleate and 1-chloro-2,4-dinitrobenzene [155], as well as environmental pollutants such as respirable neat petroleum diesel and 50% biodiesel/diesel blend [156]. In addition, environmental factors that inhibit the activity of antioxidant enzymes, such as fluoride, are also known to induce glutathione depletion in human spermatozoa and a corresponding loss of sperm function [157].

## 3. Capacity of Spermatozoa to Repair Oxidative Damage

As well as the intrinsic lack of intracellular antioxidant protection, human spermatozoa are also characterized by a limited repertoire of repair mechanisms. In terms of lipid peroxidation pathways, we have already mentioned the particular importance of aldehyde dehydrogenase [63]. Phospholipase A2 (PLA2) is also critical because this enzyme cleaves oxidized fatty acids from the second position of membrane phospholipids so that they can be neutralized by glutathione [158].

ROOH + 2GSH = ROH + GSSG + H_2_O

Lipid peroxide reduced glutathione oxidized glutathione

The lysophospholipid created by PLA2 action then has to be repaired by a reacylation reaction catalysed by lysophospholipid acyltransferase, which is a known constituent of human spermatozoa [159]. In addition, spermatozoa possess a form of phospholipid hydroperoxide glutathione peroxidase that can detoxify membrane lipid peroxides in situ, without the need for prior PLA2 action [160]. Moreover, the activity of this form of GPx is known to be deficient in the spermatozoa of infertile men [161]. Another molecule that is known to possess both PLA2 and peroxidase activities is peroxiredoxin 6, which is thought to represent a first line of defence against oxidative stress in human spermatozoa [162]. In knockout mouse models, Prdx6-/- spermatozoa display low motility and severe DNA damage, which impairs the fertilizing capacity of the spermatozoa in vitro and reduces litter size in vivo when compared to wild-type controls [163].

In addition to protecting themselves against the damaging impact of lipid peroxidation, it is clearly critical that these cells also have some capacity to deal with oxidative DNA damage when this affects the sperm nucleus. Sperm DNA is partially protected from damage by virtue of its highly condensed state, achieved via the extensive replacement of nuclear histones with small basic proteins, protamines, that neutralize the negative changes present on the DNA backbone to permit a high level of compaction. In addition, human sperm protamines contain cysteine residues that promote the further condensation of the sperm chromatin via the formation of extensive inter- and intra-molecular disulphide, and rarely, cysteine-tyrosine, bridges [164]. However, this strategy is not completely effective because human spermatozoa have been recognized for some time to contain high levels of oxidative DNA damage [28,165,166]. Spermatozoa address the formation of oxidatively-damaged DNA with a severely truncated base excision repair pathway which is initiated by OGG1 (8-oxoguanine glycosylase) [167]. This enzyme achieves the excision of 8-oxoguanine from the DNA, leaving an abasic site. It is demonstrably active in human spermatozoa because, under conditions of oxidative stress, 8-oxoguanine is actively cleaved from the sperm nucleus resulting in the appearance of this base adduct in the extracellular space [167]. In the canonical base excision repair pathway, the action of OGG1 would be followed by APE1 (Apurinic/apyrimidinic Endonuclease 1), which creates 3′-OH termini on the phosphodiester backbone in order for polymerases to insert an unmodified nucleotide, and ligase III to seal the nicks on the 3′ and 5′ ends of the backbone, with XRCC1 (X-ray repair cross-complementing protein 1) playing a scaffolding role. Human spermatozoa possess neither APE1 nor XRCC1, and so the base excision repair pathway stalls at this point. As a consequence of such limited repair capacity, an oxidative attack on human spermatozoa will result in a nucleus containing either basic sites or unresolved 8-oxoguanine residues that, if fertilization occurs, will subsequently have to repaired in the time that elapses between sperm–oocyte fusion and S-phase of the first embryonic cleavage division. If this repair is inadequately performed, it may lead to the creation of de novo mutations that may impact the health and wellbeing of the offspring [168].

## 4. Conclusions

**Figure 3:**
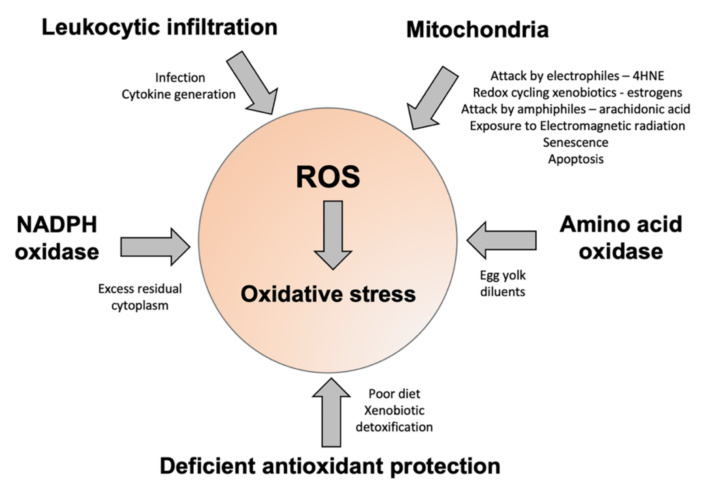
A summary of some of the factors contributing to oxidative stress in human spermatozoa. The major sources of ROS include disturbed electron flow in the sperm mitochondria, enhanced amino acid oxidase activity, or an increase in ROS generation by NADPH oxidases such as NOX5. Some of the drivers for enhanced ROS generation via these pathways are highlighted. Oxidative stress can also arise because of deficiencies in antioxidant protection due to impaired intake in the diet, or high levels of antioxidant turnover/consumption due to chronic ROS generation, associated with conditions such as obesity, varicocele, and the detoxification of environmental toxicants.

In summary, there is little doubt that oxidative stress is a major contributor to male and female infertility [169,170]. Moreover, such stress can have a major impact on the health and wellbeing of future generations as a result of both epigenetic and genetic damage to the germ line that is oxidatively induced. Space does not permit a detailed examination of all these effects; however, a number of authoritative reviews on these elements of the oxidative stress equation already exist [13,171,172,173]. In the male, it is clear that such stress can arise via a range of different mechanisms, including the infiltration of leukocytes in response to pro-inflammatory signals generated within the male tract, the generation of ROS by the sperm mitochondria, and the aberrant regulation of enzymes (NADPH and amino acid oxidases) that normally serve to provide the redox drive to sperm capacitation (Figure 3). Ultimately, whether or not the excessive generation of ROS from these sources leads to a state of oxidative stress depends on the balance of pro- and antioxidant forces prevailing during germ cell development. In this context, the adequacy of the patient’s antioxidant defences may be as important as the excessive generation of ROS in determining the overall redox balance. In order to address this problem, antioxidant supplementation has been assessed in the context of both male and female infertility, although, sadly, such trials have been flawed by a lack of diagnostic rigour [174]. In a vast majority of trials, antioxidants have been given to infertility patients irrespective of whether they are suffering from oxidative stress. The field is currently in urgent need of a simple diagnostic test that can be used for routine diagnostic purposes. Preferably, such tests should provide feedback on levels of lipid peroxidation in the blood and semen, as well as the extent of oxidative DNA damage in the spermatozoa. Although sophisticated laboratory-based techniques exist to generate such information, there is also a need to develop simple procedures that can be applied in the clinic to determine who is actually suffering from oxidative stress and, therefore, who might benefit from antioxidant intervention.

Giving random antioxidants, or antioxidant formulations in arbitrary doses, to patients with no prior knowledge of the status or levels of oxidative stress, runs the serious risk of inducing a state of reductive stress [12,170], which is potentially equally harmful to the functional competence of spermatozoa. Indeed, it is worth recalling that the redox imbalance that initiates many oxidative stress pathways often commences with reductive stress in the sense that it is the reduced component of the redox couple that is in abundance (NADH, NADPH, Fe2+ etc). In other words, at the fountainhead of oxidative stress, it is often a surfeit of electrons that is the problem, not a deficit. Via a variety of pathways, these surplus electrons are swept up by oxygen to generate ROS and activate the oxidative stress pathways leading to male infertility and DNA damage. In cases where reductive stress is at the heart of the condition, the nature of any antioxidant supplementation needs to be carefully considered to avoid exacerbating the condition, rather than resolving it. Thus, a knowledge of the pathways involved in the generation of ROS and the induction of oxidative stress, as set out in this review, may not only be valuable in determining which cases should receive antioxidant therapy, but may also help define the type and dose of antioxidants that might be the most beneficial.
